# What Psychoanalysis, Culture And Society Mean To Me

**DOI:** 10.4103/0973-1229.32159

**Published:** 2007

**Authors:** Lynne Layton

**Affiliations:** *Editor, Psychoanalysis, Culture and Society, An analyst in private practice in Brookline, Massachusetts, USA. BIDMC Department of Psychiatry, Harvard Medical School, USA. E-mail: layton@fas.harvard.edu*

**Keywords:** *Social Psychoanalysis*, *Psychoanalysis*, *Culture*, *Society*, *Social Hierarchies*, *Psychic Resistance*, *Challenge to Social Norms*, *‘Normative Unconscious Processes’*

## Abstract

The paper reviews some ways that the social and psychic have been understood in psychoanalysis and argues that a model for understanding the relation between the psychic and the social must account both for the ways that we internalize oppressive norms as well as the ways we resist them. The author proposes that we build our identities in relation to other identities circulating in our culture and that cultural hierarchies of sexism, racism, classism push us to split off part of what it means to be human, thereby creating painful individual and relational repetition compulsions. These “normative unconscious processes” replicate the unjust social norms that cause psychic pain in the first place. The paper concludes with thoughts about contemporary US culture, in which the government has abdicated responsibility toward its most vulnerable citizens and has thus rendered vulnerability and dependence shameful states.

## Introduction

As editor (with Simon Clarke) of the journal *Psychoanalysis, Culture and Society (http://www.palgrave-journals.com/pcs/index.html)*, I am constantly made aware of the multiple ways in which theorists and clinicians conceive of the relation between the unconscious and the social world. The relation between the two was on analysts’ minds from the earliest days of psychoanalysis, and even then there were multiple ways of interpreting that relation. In the realm of practice, Danto (1998) writes that Freud felt psychoanalysis ought to be available to and would be useful to the most oppressed segments of society as well as to the rich. Freud thus gave his imprimatur to the free clinics in Berlin and Vienna set up in the 1920′s. Addressing a different aspect of the relation between the psychic and the social, Freud (1908/1959) wrote about the ways that a sexually repressive society could cause neurotic anxieties, and Fenichel (1953) wrote about the relation between capitalist culture and anal neuroses. In addition to Fenichel, Wilhelm Reich, members of the Frankfurt School (Fromm, Adorno, Marcuse) and others contributed to what was to become a long tradition of theories that conceive of character conflicts as precipitates of socio-economic structures and contradictions-more recent examples in this tradition are Christopher Lasch's (1979) *The Culture of Narcissism* and Tod Sloan's (1996) *Damaged Life*.

In most of his works on psychoanalysis and culture, Freud argued that the psyche-social relation also operates in the opposite direction, perhaps fundamentally so. In *Totem and Taboo* (1913,1955), *Moses and Monotheism* (1939), *Group Psychology and the Analysis of the Ego* (1922; 1959), he put forth his primal horde theory, in which the sons’ rage at the primal father's freedom to exercise his sexual and aggressive instincts without restraint leads them to murder the father. The ensuing sense of guilt ushers in a more egalitarian, but at the same time a far more repressed version of society, in which no one is allowed to enact the primal father's instinctual freedom. In this model, neurotic conflicts are primary in determining the form of a society, whereas in the model described earlier, society is the prime cause of neurotic conflicts.

## Internalisation of Norms, Psychic Resistance and Challenge to Social Norms

The models I've laid out thus far tend to select either the social or the psychic as their prime mover. It seems to me that the greatest challenge for those of us theorizing about psychoanalysis, culture and society is to resist the either/or and to derive a model that neither reduces the psychic to the social nor reduces the social to the psychic. As I have argued elsewhere (2004a), the problem for a social psychoanalysis is to account both for the ways that norms are internalized as well as for what makes psychic resistance and challenge to social norms possible.

My own way of conceiving such a model draws on a variety of sources, psychoanalytic and non-psychoanalytic: Foucaultian ideas of knowledge-power (1982); psychoanalytic versions of performance theory (Butler, 1995); Bourdieu's (1984) description of the unconscious development of and libidinal attachment to one's habitus; psychoanalytic theories of psycho-social character formation and repetition compulsion; interpersonal/relational analytic theory, particularly its feminist and left-wing articulations; Gramsci's (1971) theory of hegemony and elaborations of that theory by Hall (1980) and by Laclau and Mouffe (1985); Kleinian (1946;1986) ideas regarding splitting and projective identification; Kohut's (1971, 1977) theory of narcissism; and a burgeoning recent literature on what causes clinical impasses and how one extricates oneself from them (see, for example, Stern, 2004).

I began articulating what psychoanalysis, culture and society mean to me in my book *Who's That Girl? Who's That Boy? Clinical Practice Meets Postmodern Gender Theory* (1998, reprinted in 2004b). My concern there was not only to understand the way that the internalization of sexist gender norms produces various forms of psychic pain for men and women, but also to bring into conversation two disparate discourses on gender: a poststructuralist academic feminist discourse that largely argued that all forms of identity subjugate the subject and an object relations and relational psychoanalytic feminist discourse. I argued that post-structuralist accounts offered psychoanalytic theorists a way of thinking about power differentials and the constricting aspects of what society posits at any given time as ideal versions of femininity and masculinity; poststructuralism well articulates the coercive aspect of social norms and how they set limits on what is psychically possible. At the same time, I also argued that identities and identifications are multiple, conscious and unconscious and not simply imposed in any linear fashion. One develops one's gender identity and, indeed, one's subjectivity within an overlapping and contradictory assortment of relational matrices, and the idiosyncratic and unpredictable way that one makes meaning of one's relational interactions guarantees that there is no way to predict how social norms will be internalized and expressed. Social movements such as feminism, gay liberation and black power demonstrate the way that identities can be deployed in such a way as to facilitate growth. At the same time, however, hierarchies of gender, race, class and sexuality do make certain types of internalizations prevalent. Thus, identities and identifications must be understood in both their potentially traumatizing as well as their potentially liberating forms.

Drawing on the analytic theory of narcissism developed by both Kohut (1971, 1977) and Kernberg (1975), and the relational-conflict theory of Mitchell (1988), I suggest that one's relational repertoire derives from one's own particular way of making meaning of and interweaving experiences that have been narcissistically wounding, traumatizing and humiliating, with experiences in which one has felt recognized and acknowledged as a subject who has his/her own separate thoughts, feelings, wishes, etc. My sense, then, is that our subjectivity, identities and identifications are “negotiated” (Pizer, 1998) out of two disparate kinds of relationships: those that Jessica Benjamin (1988, 2004) has called doer-done to relations, in which we are not recognized in all our complexity and are treated as objects rather than subjects; and those in which we are recognized as interdependent but separate subjects, with our own agendas.

### Social Hierarchies Pull for Splitting

The problems that I see as both social and individual problems are often the sequelae of the way that hierarchies of gender, race, class, sexuality operate in any given culture. It is my sense that social hierarchies are most psychologically damaging because they require a splitting of human capacities (between genders, races, classes) that, in health, ought to be integrated rather than split. For example, when I was growing up in the 50s and 60s, a middle-class white female in the US generally received love and approval for developing and exercising her relational and caretaking capacities (see Chodorow, 1978). Ambition and assertive strivings were allowed only insofar as they did not threaten relational priorities; if they did, they were coded “bad,” unfeminine. To this day, when I am at a weekend family gathering and need to take some time to read a book or prepare a class assignment, I am aware of the disapproving glances from the matriarchs chatting at the kitchen table. The men, who can generally be found watching a football game or doing any number of mildly or completely non-relational activities-retreating to a private space to work on or play with a computer, for example-do not receive such glances. It is the damning glances, harsh words and withheld love that penetrate the psyche and that, when repeated over time, push one to split off parts of oneself in order to get or retain love and approval.

In *Who's That Girl*, I argued that the regime of white middle-class normative ideals of femininity and masculinity in which I grew up in the 50s-heir to the gendered public/private split in 19^th^ century US life that was temporarily eased during WWII but starkly reinforced in the post WWII era-pulled for two subtypes of narcissism. In the female version, in which one receives love for developing one's relational capacities, assertiveness might be at best toned down, at worst split off and projected outwards, dissociated, disavowed. But it doesn't disappear. Rather, it takes on subterranean and symptomatic forms, such as nagging and body complaints. Meanwhile, the version of attachment that remains is also marked by the trauma of having to repudiate part of what it means to be human. When assertiveness and attachment are split and gendered, it is not possible for women to be models of healthy relationality, as some gender theories of the 1980′s, influenced by Gilligan (1982), maintained. Deprived of permission to be assertive, many women of my generation developed angry or hostile-dependent attachments and deprecating attitudes towards the self. In this subtype of narcissism, women were likely to project agency into men and have difficulty experiencing the self as a separate center of aliveness and agency. Kohut (1971) would have described the narcissistic gratification as follows: you (the male subject to whom a heterosexual female attaches) are perfect and I am part of you. In the case of the middle-class male normative ideal, one receives love and approval for agentic strivings, but vulnerability, dependency and emotionality are split off and repudiated as “feminine” (see Chodorow, 1978). The version of agency thus produced is another subtype of narcissism: shamed for feeling vulnerable, emotional and dependent; men were pulled to develop a grandiose and dominating sense of self, one in which the other is devalued and the need for the other is denied. Thus do psychological structures such as dependency, agency, vulnerability, reason, etc. become gendered, raced, classed and sexed.

The way they become so differs, of course, in different cultures and subcultures and in different historical moments. To understand the way cultural hierarchies influence the development of subjectivity in any given culture, researchers must carefully attend to local and historical specificity. Nonetheless, I think it a universal principle that the stuff of psychic life-negotiating dependency, autonomy, affect, needs for recognition and love-is influenced by the way individuals psychically negotiate, consciously and unconsciously, the interplay between the norms of dominant culture and those of local and subordinate cultures. Race, class, caste, gender, sexual, religious and other cultural norms intersect with each other in contradictory and complementary ways, as I will describe later. And we build those intersectional identities always in relation to the other identities circulating in our culture. In this globalized and mass-media saturated world, we now build our identities in relation to identities circulating in other cultures as well. This can have both constricting and liberating effect, sometimes both at once. In the US, white youths “use” black culture to contest constricting white norms and, in many cases, that use creates the possibility of breaking down racist barriers to intergroup intimacy. The influence of US popular culture abroad often creates new social problems (for example, eating disorders among women whose cultures introduce US television and its norms of female beauty), but at other specific times and places, it has offered liberatory possibilities (for example, the way some Eastern and other Western youth cultures used US and UK popular music in the 60s and 70s to protest coercive norms in their own cultures and as a basis for their own indigenous music).

### ‘Normative Unconscious Processes’

In my own work, as I thought further about the coercive effects that hierarchies of race, class, gender and sexuality have on subjectivity, I developed the concept of “normative unconscious processes” to account for the way that norms are conflictually internalized and expressed/elaborated. Because social hierarchies split and categorize human attributes and capacities, because they enforce a kind of doer-done to relation in which approval is given only for performing gender or race “properly,” we find, in the clinic and in our lives, unceasing conflict between those unconscious processes that seek to maintain the splits and those that refuse them. The ones that seek to maintain the splits are those that I call “normative unconscious processes,” and these processes pull us to repeat affect/behaviour/cognition patterns that uphold the very social norms that cause psychic distress in the first place. In several of my papers (2002, 2004c, 2006a), I have given examples of how patient and therapist collude in normative unconscious enactments that perpetuate racism, sexism, heterosexism and class inequality. It has become one of my main interests to understand how what I know about the interplay between psyche and society plays itself out in the unconscious dynamics of patient and therapist in the clinic. In the history of psychoanalytic theorizing, even within that small body of work that does investigate the relation between psyche and society, clinical applications have been a fairly neglected research domain.

In one example of enactments of normative unconscious processes (Layton, 2006b), I discussed the pull on the patient and therapist alike to uphold the US cultural norm that insists we separate the psychic from the social. In my experience, most of the dominant discourses in US life, including most psychoanalytic theories and practices, sustain the fantasy that the two are separate. Many are the times that my work has been criticized for “importing” politics into the supposedly apolitical realm of psychoanalysis. My reply is that everything is political (Samuels, 1993) and that what seems apolitical only seems so because the mainstream is in the position to call its own views “neutral” and views on the margin “political.” In a co-edited book on class and politics in psychoanalysis (Layton, Hollander and Gutwill, 2006), a group of like-minded analysts countered the trend that splits the psychic and the social in psychoanalytic work. My own contribution (2006b) described work with a patient who brought in a dream about a politician and then became uncomfortable when her associations led her to talk about her politics. Both she and I discovered a resistance to letting the political in; we both felt a strong pull to interpret the content of the dream in terms of early family conflict, a pull that interfered with and nearly shut down a passion rarely glimpsed in this patient-indeed, her lack of passion was one of her primary complaints on entering therapy.

As I said above, within mainstream psychoanalysis it is still controversial to consider culture as a mediating influence in the development of the psyche. As mainstream psychiatry in the US has become more and more captive to what I would regard as a reductionist version of science, “scientism,” psychoanalysis has been increasingly marginalized. I went to psychology graduate school in the early to late 80s and at that time my department was psychoanalytic, as was the hospital in which I did my internship. Currently, my graduate programme focuses mainly on neuroscience and cognitive behavioral paradigms, the psychology training programme was eliminated from the hospital, and the criteria for advancement at the medical school at which I work discriminate against those who do psychoanalysis rather than “science.” My own work is, in these quarters, considered sociology and not psychiatry. Unfortunately, sociology is not terribly open to psychoanalysis either, and there is nothing in the US that approximates the psycho-social studies programmes that exist, for example, in the UK. Psychoanalysis in the US thrives predominantly in literature departments (which largely consider only Freud and Lacan) and in psychoanalytic institutes, where, ironically, there has been an explosion of new theories and models at the very moment when psychoanalysis has lost its cultural cachet and its professional dominance in psychiatry departments.

## Media, Unconscious Fantasies and Normative Unconscious Processes

As it happens, before becoming a psychologist and psychoanalyst, I was trained as a literary critic and later taught in the area of popular culture/cultural studies. I shall conclude by discussing some of my work in this area, another arena in which I investigate the relation between the psychic and the social. Much of the work in cultural studies focuses on the way media create identifications, structure subjectivities and pull for particular kinds of unconscious fantasies that, as with the normative unconscious processes I discussed above, tend to affirm a capitalist or a racist or a sexist status quo. In trying to understand what has been going on politically and psychically in the US during the right wing backlash that has dominated the past six years, I have been most struck by the way that need, dependency and vulnerability have become increasingly marked as shameful-at the same time that fears about an out of control world are daily stoked by media and government alike.

When I think about the way such vulnerabilities are expressed in the clinic, one of the few places left in the US where one has permission to express them without shame, I find that, for many people, the two primary defenses against exposure of vulnerability are either withdrawal or retaliation. While I do not think that one can apply concepts of individual psychology to group psychology in any linear fashion, I do think that one thing we are witnessing in the US today is what I have elsewhere called (Layton 2006c) a politics of attack and withdrawal. The politics of attack are clearly seen in the virulent and hate-filled backlash against the gains made by the social movements of the last half of the twentieth century: civil rights, feminism, gay liberation, anti-racist movements of all kinds. The politics of withdrawal are seen in the general apathy of the voting population, but most troublingly perhaps in privileged, liberal circles. In Rachael Peltz's “*The Manic Society*” (2006), she argues that, dating from the Reagan years and the conservative attack on “big government,” the US government scaled back its commitment to public welfare in general and to its most vulnerable citizens in particular. Peltz talks about the absence of a containing function of contemporary government, and she argues that this promotes a culture in which one manically denies one's need for the other and for caretaking. The ethos that global capitalism and both neoconservative and neoliberal ideologies have promoted since the late 1970s is an ethos of “save your own skin”; and I think we see this everywhere, from sadistic and humiliating reality TV shows where only one last “winner” survives, to the consulting room where, as I said earlier, dependency is generally felt to be shameful and interdependence is a concept few can acknowledge.

### Clinical Theory and Practice, Cultural Interdependence

In what ways might our clinical theory and practice collude with a culture that promotes a terrified and terribly lonely version of independence? While I certainly think that psychoanalytic practitioners value interdependence as an ideal of mental health, I also think that the fact that we exclude cultural considerations from the clinic forces upon us perhaps a too narrow vision of interdependence, one limited to the private sphere of family and close friends. So what I have begun to think about, drawing again on contemporary cultural theory and art, are the ways in which we are mutually implicated in each other's lives, joys and pains. As I said earlier, if cultural hierarchies enforce splitting, and splitting requires that we keep near what we split off, then it seems to me that our identities are always built in relation to other identities circulating in our particular culture, and our own investments in gender, race, class and sex must be implicated in the investments of those we consider “other.” Beverley Skeggs's study (1997) of a group of British working-class women who seek upward mobility by enrolling in care-giving training programmes offers a good example of what I am theorizing. Skeggs describes the way that these women create their identities in opposition to the upper class women for whom they work, claiming a kind of respectability for themselves based on what they consider to be their superior capacities to give care, capacities they find wanting in their self-absorbed, always busy female employers who “shop out their kids” (p. 71). At the same time, for Skeggs's subjects, becoming respectable also means dis-identifying with the ways the female working class subject is typically figured in upper-class valuations-as slutty, less moral in general and wanting in sophistication and taste. In the norms of dominant culture, it is the middle class female who holds the claim on respectability that this particular group of working class females covet and attempt to re-define in their favour. Yet, as Skeggs shows, they never quite feel capable of securely possessing respectability.

Other examples of the way social and political norms affect the way identities are lived in relation to other identities can be found in the 2002 anthology, *Global Woman* (Ehrenreich and Hochschild), which focuses on what the editors call a “care drain” (vs. a brain drain) from Third World to First World countries. The book's essays provide interesting sociological data on the transfer of domestic services from low-to high-income cultures. Chapters document, for example, the toll taken on the children left behind by mothers who emigrate in hopes of improving their families’ welfare, women who end up being nannies and taking care of First World women's children. The mothers’ pain, their children's pain, their double-binding love for the children they care for, their resentments toward their employers-all beg for psychoanalytic study (see the special issue of *Studies in Gender and Sexuality*, 2006, in which psychologists and psychoanalysts discuss the book)- but too often psychoanalysis and sociology are kept separate in the US, to the detriment of each.

These examples of the way that subordinate and dominant identities are built in relation to one another also exemplify the way that, within a given culture, we become implicated in each other's vulnerabilities and pain. That part of identity that one uses defensively to, in Bourdieu's words, achieve distinction, is the very part that one learns to wield against others (Bourdieu, 1984). Again, I see this as a dominant psychic outcome of forming one's identity in accord with norms that sustain cultural hierarchies by requiring that we can only attain the “desirable” identity if we split off part of what it means to be human. We are then forever vulnerable to sudden exposure of that part of us that threatens to “unseat” us from the throne of distinction. As I think more about how, for example, one group's repudiation of dependency might psychologically affect those lower on the social totem pole, those who are called upon to shield those above from knowing how dependent they are, I become aware of the fact that, as Butler (2004) has said, it is our vulnerabilities that bind us one to the other-both in joy and pain. How a culture manages vulnerability, what defenses against it are promoted or discouraged; how a culture divides up its resources and to which of its human subjects it gives recognition and from which subjects it withholds it; how big a divide exists between rich and poor, deserving and undeserving and what forms of “distinction” rise up to deal with these inequities-these are some of the social variables that affect what we see and do in the clinic.

## Concluding Remarks

In conclusion, I am arguing that many of the psychological problems that clinicians treat are a result of social inequalities. While identity categories can be deployed in ways that facilitate growth, social inequalities such as sexism, racism and classism cause wounds that split the psyche, creating shameful vulnerabilities that we defend against by wielding identity as a weapon against others. In my view, it is both difficult to recognize and to acknowledge the fact that we are all mutually implicated in each other's identity wounds, that we are both victims of inequality and perpetrators of it. Until we are able to do this, however, both in the clinic and in the culture at large, we will continue to perpetuate conditions of inequality.

### Take Home Message

The process of identity formation in social conditions of inequality results in the splitting off of part of what it means to be human. As all identities are formed in relation to other identities circulating within a culture, the splits implicate us in each other's suffering.

## Questions That This Paper Raises

- How do we account for the intersection of society and individual subjectivity?
- In what ways are societal norms lived?
- What do we mean by a social unconscious or by normative unconscious processes?
- How do unconscious processes operate in identity formation?
- What effects do cultural hierarchies and unjust social norms have on the psyche?
- What effects do political events and the media have on group psychological processes?

## About the Author

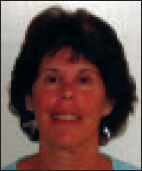



Lynne Layton, Ph.D, is Assistant Clinical Professor of Psychology, Harvard Medical School. She has taught culture and psychoanalysis for Harvard's Committee on Degrees in Social Studies and teaches at the Massachusetts Institute for Psychoanalysis. She is the author of Who's That Girl? Who's That Boy? Clinical Practice Meets Postmodern Gender Theory (Analytic Press, 2004), co-editor, with Susan Fairfield and Carolyn Stack, of Bringing the Plague. Toward a Postmodern Psychoanalysis (Other Press, 2002) and co-editor, with Nancy Caro Hollander and Susan Gutwill of Psychoanalysis, Class and Politics: Encounters in the Clinical Setting (Routledge, 2006). She is Editor of Psychoanalysis, Culture and Society and associate editor of Studies in Gender and Sexuality. Her private practice of psychoanalysis and psychotherapy is in Brookline, Ma, USA.

## References

[1] Benjamin J (1988). The Bonds of Love.

[2] Benjamin J (2004). Beyond Doer and Done-To: An Intersubjective View of Thirdness. Psychoanalytic Quarterly.

[3] Bourdieu P (1984). Distinction.

[4] Butler J (1995). Melancholy Gender—Refused Identification. Psychoanalytic Dialogues.

[5] Butler J (2004). Precarious Life. The Powers of Mourning and Violence.

[6] Chodorow N (1978). The Reproduction of Mothering.

[7] Danto EA (1998). **The** Ambulatorium: Freud's Free Clinic in Vienna. International Journal of Psychoanalysis.

[8] Ehrenreich B, Hochschild A (2002). Global Woman. Nannies, Maids, and Sex Workers in the New Economy.

[9] Fenichel O, Fenichel H, Rappaport D (1953). The Collected Papers of Otto Fenichel.

[10] Foucault M (1982). The Subject and Power. Critical Inquiry.

[11] Freud S, Jones E (1908/1959). ‘Civilized’ Sexual Morality and Modern Nervousness. Sigmund Freud. Collected Papers.

[12] Freud S, Strachey J (1913,1955). Totem and Taboo. The Standard Edition of the Complete Psychological Works of Sigmund Freud.

[13] Freud S, Strachey J (1922/1959). Group Psychology and the Analysis of the Ego.

[14] Freud S (1939). Moses and Monotheism.

[15] Gilligan C (1982). In a Different Voice. Psychological Theory and Women's Development.

[16] Gramsci A, Hoare Q, Smith GN (1971). Selections from the Prison Notebooks.

[17] Hall S, Hall S, Hobson D, Lowe A, Willis P (1980). Encoding/Decoding. Culture, Media, Language Working Papers in Cultural Studies 1972-1979.

[18] Kernberg O (1975). Borderline Conditions and Pathological Narcissism.

[19] Klein M, Mitchell J (1946/1986). Notes on Some Schizoid Mechanisms. The Selected Melanie Klein.

[20] Kohut H (1971). The Analysis of the Self.

[21] Kohut H (1977). The Restoration of the Self.

[22] Laclau H, Mouffe C (1985). Hegemony and Socialist Strategy.

[23] Lasch C (1979). The Culture of Narcissism.

[24] Layton L (1998/2004). Who's That Girl? Who's That Boy? Clinical Practice Meets Postmodern Gender Theory.

[25] Layton L, Fairfield S, Layton L, Stack C (2002). Cultural Hierarchies, Splitting, and the Heterosexist Unconscious. Bringing the Plague. Toward a Postmodern Psychoanalysis.

[26] Layton L (2004a). A Fork in the Royal Road: Defining the “Unconscious” and its Stakes for Social Theory. Psychoanalysis Culture & Society.

[27] Layton L (2004b). That Place Gives Me the Heebie Jeebies. International Journal of Critical Psychology. Psycho-Social Research.

[28] Layton L (2006a). Racial Identities, Racial Enactments, and Normative Unconscious Processes. Psychoanalytic Quarterly.

[29] Layton L, Layton L, Hollander NC, Gutwill S (2006b). Attacks on Linking. Psychoanalysis, Class and Politics: Encounters in the Clinical Setting.

[30] Layton L (2006c). Retaliatory Discourse: The Politics of Attack and Withdrawal. International Journal of Applied Psychoanalytic Studies.

[31] Layton L, Hollander N, Gutwill S (2006). Psychoanalysis, Class and Politics: Encounters in the Clinical Setting.

[32] Mitchell SA (1988). Relational Concepts in Psychoanalysis.

[33] Peltz R, Layton, Hollander, Gutwill (2006). The Manic Society. Psychoanalysis, Class and Politics: Encounters in the Clinical Setting.

[34] Pizer R (1998). Building Bridges. The Negotiation of Paradox in Psychoanalysis.

[35] Roundtable on Global Woman (2006). Studies in Gender and Sexuality.

[36] Samuels A (1993). The Political Psyche.

[37] Skeggs B (1997). Formations of Class and Gender: Becoming Respectable.

[38] Sloan T (1996). Damaged Life. The Crisis of the Modern Psyche.

[39] Stern D (2004). The Eye Sees Itself: Dissociation, Enactment, and the Achievement of Conflict. Contemporary Psychoanalysis.

